# Mugifumi, a beneficial farm work of adding mechanical stress by treading to wheat and barley seedlings

**DOI:** 10.3389/fpls.2014.00453

**Published:** 2014-09-12

**Authors:** Hidetoshi Iida

**Affiliations:** Department of Biology, Tokyo Gakugei UniversityKoganei-shi, Tokyo, Japan

**Keywords:** mechanical stress, mechanosensing, treading, trampling, stamping, wheat, barley, crop yield

Plant scientists are now aware that mechanical stresses, such as touching, bending and treading, affect the growth and development of trees and grasses, including crops (Jaffe, 1973; Mitchell and Myers, 1995; Telewski, 2006). However, Japanese farmers have known for centuries of the efficacy of applying a mechanical stress—treading, trampling, or stamping—to the seedlings of autumn-sown wheat and barley, and call this process “mugifumi” [mugifúmi] in Japanese. According to a comprehensive, narrative guidebook written in the 17th century, Japanese farmers enthusiastically treaded seedlings in winter because they empirically knew that treading prevented spindly growth, strengthened the roots to grow and spread, shortened plant height, increased tillers and ear length, and eventually gave a good yield (Anonymous, 1680's). Japanese researchers at agricultural experiment stations have described the methodology of treading and many quantitative data on its remarkable effects over the last several decades. However, the information on this practice in English has remained scarce, since it had been targeted to local farmers and written in Japanese only. I here attempted to provide an insight into the significance of treading and advocate the importance of further studies with cutting-edge technology to promote a better understanding of the molecular mechanisms underlying mechanosensing and mechanotransduction in crop plants.

## What is mugifumi?

**Figure 1:**
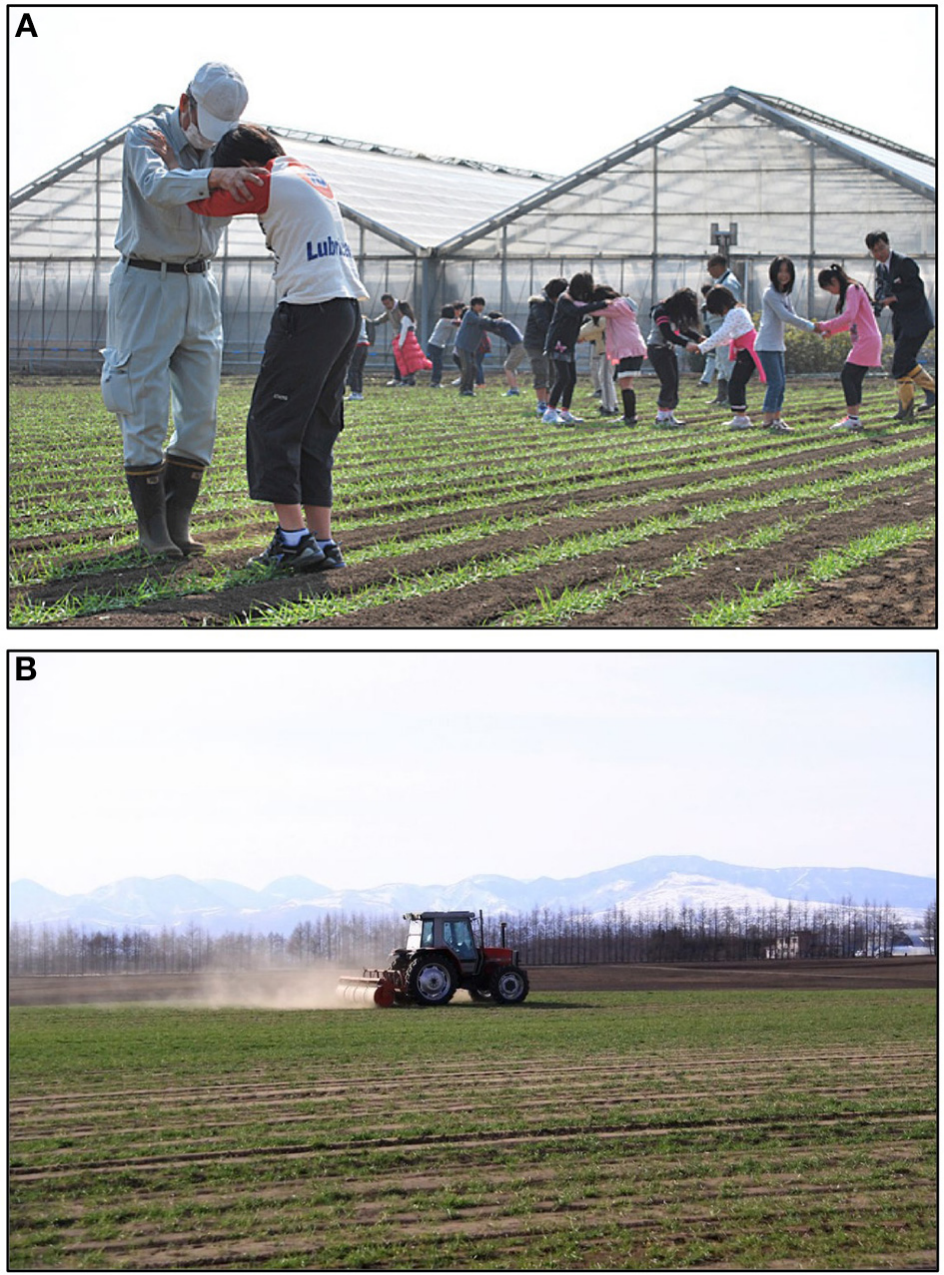
**Scene of mugifumi. (A)** Are they dancing in the wheat fields? No, they are not. Farmers and their neighboring children tread wheat seedlings (in Fujisawa, Kanagawa Pref., Japan). Courtesy of the Chiikimiryoku, a nonprofit organization. **(B)** Large-scale treading. Farmers use an agricultural tractor equipped with a specifically designed treading roller to tread wheat seedlings (in Kamishihoro, Hokkaido, Japan). Courtesy of Mr. Haruo Hoshino.

Mugifumi is the combination of mugi and fumi. Mugi is the general term for wheat and barley and fumi means treading. Therefore, mugifumi is the act of treading wheat and barley plants, especially their seedlings. In relatively small fields, farmers, their families, and sometimes neighbors tread the seedlings using their feet (Figure 1A), while farmers use an agricultural tractor equipped with a treading roller to tread seedlings in large fields (Figure 1B). Mugifumi is also called “touatsu” [touátsu], which is the combination of tou (treading) and atsu (pressure). Mugifumi is more familiarly used than touatsu.

Mugifumi is known to significantly affect wheat and barley plants. Treated plants have less spindly growth and lodgings, more tillers, longer spikes, and higher yields than untreated plants if farmers perform mugifumi properly.

The timing and number of times mugifumi is performed are important to obtain good results. Special care is needed when performing mugifumi because the growth of wheat and barley varies from year to year and from region to region. This variation depends on wheat and barley varieties and is influenced by yearly and weekly climate changes. If farmers miss the right time and perform mugifumi an incorrect number of times, they may damage the seedlings and have a reduced grain yield. The appropriate weight for mugifumi corresponds to the body weight per seedling.

Wheat and barley seeds are generally sown in the period between November and early December in Japan. Treading is typically started early in January when the seedlings have three leaves, is then performed several times almost every 10 days, and is stopped before internodes start to grow. The number of times treading is performed varies depending on the growth conditions. To avoid letting the soil become too firm, it is important to tread seedlings when the soil is dry. Another beneficial effect of treading is that it permits roots that have risen from the soil due to the growth of frost columns to be settled.

## How effective is mugifumi?

Although many studies have been written in Japanese on the effects of mugifumi on the growth of wheat and barley plants and the yield of gains, to the best of my knowledge, none have been written in English. Of those written in Japanese and available online, a small section of the most comprehensive article written on mugifumi by Ohtani (1950) has been described herein. This article covered a wide range of effects of mugifumi on wheat and barley, including those on developmental, morphological, and physiological traits. I focused on some developmental traits of treaded wheat plants, including grain yields.

Supplementary Table 1 shows the effects of treading on the development of wheat seedlings. In this experiment, the seeds of wheat were sown in a field in the middle of November; seedlings were treaded with feet 32, 62, 73, and 84 days after sowing; and the effects of treading were examined 101 days after sowing. Treading clearly resulted in favorable effects on seedling development from an agricultural viewpoint. Regarding the shoot, the numbers of stems and leaves as well as the wet weight were increased by treading by 74, 10, and 50%, respectively. The length and number of roots were also increased by 4 and 7%, respectively. These findings indicate that treading induces tillering and stimulates the roots to grow and spread. These quantitative findings substantiate the accuracy of the narrative guidebook for farmers described over 300 years ago (Anonymous, 1680's).

Supplementary Table 2 shows the effects of wheat treading on grain yields. In this experiment, seeds were sown on seedbeds near the end of October and the seedlings were transplanted to fields 42 days after sowing. Treading was performed twice on seedlings with feet 151 and 156 days after sowing. This treading led to good yields: the number of spikes per plant, weight of whole plant, and grain weight per plant were increased by 18, 41, and 54%, respectively.

## Perspective

These two examples demonstrate that mechanical stress, such as treading, modifies the development and growth of wheat, resulting in higher numbers of stems, leaves, roots, and spikes, all of which increase grain yields. Although data was not shown here, a previous study reported that wounds caused by treading facilitated the evaporation of water and thus increased the osmolality of cells, thereby conferring cold resistance on the treaded seedlings (Ohtani, 1950). The effects of treading were also investigated extensively using botanical, biochemical, and physical approaches more than 60 years ago (Ohtani, 1950). However, further studies are warranted to investigate the effects of treading wheat and barley plants with modern technologies not only from agricultural viewpoints, but also from the aspect of basic science.

Beneficial effects of mechanical stress are not limited to wheat and barley. There are many studies on the effect of mechanical stress on vegetable plants (for review, see Latimer, 1991). For example, rubbed soybean plants exhibited reduction in stem elongation and frost resistance (Jaffe and Biro, 1979). Mechanically rubbed stems of common bean were hardened because of an increase in flexibility and acquired resistance to bending-caused breaking (Jaffe et al., 1984). Wind-induced mechanical stress increased resistance to an arthropod herbivore and a fungal pathogen in common bean (Cipollini, 1997).

Effects of mechanical stress on trees have also been studied extensively (for review, see Coutand, 2010). For instance, mechanical bending of the stem increased biomass allocation toward the root in wild cherry tree seedlings, avoiding the formation of poor root systems and the reduction of diameter growth of the trunk seen in mechanically untreated seedlings (Coutand et al., 2008). Mechanical stress given to the upper part of the order 1 axis of young rose plants resulted in a reduction in axis length and an increase in the number of branching, leading to the compactness of the bush, a favorable horticultural trait (Morel et al., 2012). Thus, it is possible that chemical plant growth regulators or chemical plant growth retardants, which are frequently used in horticulture, can be replaced by an appropriate mechanical stress, that is an environment-friendly treatment. As is the case for mugifumi, however, careful application of mechanical stress is needed in consideration of degree of forces applied, the stage of plant growth, soil conditions, weather, and so on. It is worthwhile to note that those favorable effects of mechanical stress on vegetable plants and trees are similarly seen on wheat and barely as described above.

As for mugifumi, the following future studies would be of interest: treading-induced early signaling, gene expression, changes in the cytoskeleton and cell wall, hormone synthesis and transport, and changes in metabolism. These researches should provide a novel insight into mechanosensing and mechanotransduction in plants, especially in monocots. Such lines of study have recently been conducted using a model dicot, *Arabidopsis thaliana*. For example, by whole genome microarray analysis, mechanical wounding of leaf tissues was shown to induce a number of rapid wound response genes that overlaps with those induced by a wide range of biotic and abiotic environmental stresses (Walley et al., 2007). Repetitive touching enhanced resistance to fungus (*Botrytis cinerea*) infection and cabbage looper (*Trichoplusia ni*) infestation in a defense phytohormone jasmonate-dependent manner (Chehab et al., 2012). Gentle mechanical sweeping of leaf surfaces induced a rapid increase in cytosolic Ca^2+^ concentration and a release of reactive oxygen species, and then enhanced resistance to *B. cinerea* in a jasmonate-independent manner (Benikhlef et al., 2013). Technically and conceptually similar studies on mugifumi-treated wheat and barley should offer a clue to increase the yield of grain and alleviate the shortage of the food worldwide without using a large amount of fertilizers, production and use of which increase environmental burdens.

### Conflict of interest statement

The author declares that the research was conducted in the absence of any commercial or financial relationships that could be construed as a potential conflict of interest.
