# Supplementary material for: Mugifumi, a beneficial farm work of adding mechanical stress by treading to wheat and barley seedlings
Source: Front Plant Sci. 2014 Sep 12;5:453. doi: 10.3389/fpls.2014.00453 (PMC4162469; doi:10.3389/fpls.2014.00453)
Supplement: Supplementary file 1 [file Table1.DOCX]

**Supplementary Table 1 Effects of treading on the development of wheat seedlings**

Height of No. of stems No. of leaves Wet weight Length of No. of roots

shoot (cm) of shoot (g) root (cm) Treaded 7.9 8.7 6.6 0.84 7.2 13.3

Untreaded 8.1 5.0 6.0 0.56 6.9 12.4

Ratio (%) 98 174 110 150 104 107

Treaded four times. Examined 101 days after sowing (*n* = 20). Adapted from Ohtani (1950).

**Reference:**

Ohtani, Y. (1950) Studies on the stamping of wheat and barley. *Bul. Natl. Agr. Exp. Stn. Jpn.* 67, 1-76 (in Japanese; Summary in English; URL, http://agriknowledge.affrc.go.jp/RN/2010826214).
