# Supplementary material for: Mugifumi, a beneficial farm work of adding mechanical stress by treading to wheat and barley seedlings
Source: Front Plant Sci. 2014 Sep 12;5:453. doi: 10.3389/fpls.2014.00453 (PMC4162469; doi:10.3389/fpls.2014.00453)
Supplement: Supplementary file 2 [file Table2.DOCX]

**Supplementary Table 2 Effect of treading wheat seedlings on grain yields**

Height of No. of spikes Weight of Grain weight

shoot (cm) per plant whole plant (g) per plant (g)

Treaded 67.6 11.7 41.1 17.4

Untreaded 67.1 9.9 29.1 11.3

Ratio (%) 101 118 141 154

Treaded twice at the stage of developing young spikes. Examined approximately 220 days after sowing (*n* = 15). Adapted from Ohtani (1950).

**Reference:**

Ohtani, Y. (1950) Studies on the stamping of wheat and barley. *Bul. Natl. Agr. Exp. Stn. Jpn.* 67, 1-76 (in Japanese; Summary in English; URL, http://agriknowledge.affrc.go.jp/RN/2010826214)
